# Advances in mapping malaria for elimination: fine resolution modelling of *Plasmodium falciparum* incidence

**DOI:** 10.1038/srep29628

**Published:** 2016-07-13

**Authors:** Victor A. Alegana, Peter M. Atkinson, Christopher Lourenço, Nick W. Ruktanonchai, Claudio Bosco, Elisabeth zu Erbach-Schoenberg, Bradley Didier, Deepa Pindolia, Arnaud Le Menach, Stark Katokele, Petrina Uusiku, Andrew J. Tatem

**Affiliations:** 1 WorldPop, Geography and Environment, University of Southampton, Southampton, UK; 2 Flowminder Foundation, Stockholm, Sweden; 3 Faculty of Science and Technology, Lancaster University, UK; 4 School of Geography, Archaeology and Palaeoecology, Queen’s University Belfast, Belfast BT7 1NN, Northern Ireland, UK; 5 Clinton Health Access Initiative, Boston, MA, USA; 6 National Vector-borne Diseases Control Programme, Directorate of Special Programmes, Ministry of Health and Social Services, Windhoek, Namibia

## Abstract

The long-term goal of the global effort to tackle malaria is national and regional elimination and eventually eradication. Fine scale multi-temporal mapping in low malaria transmission settings remains a challenge and the World Health Organisation propose use of surveillance in elimination settings. Here, we show how malaria incidence can be modelled at a fine spatial and temporal resolution from health facility data to help focus surveillance and control to population not attending health facilities. Using Namibia as a case study, we predicted the incidence of malaria, via a Bayesian spatio-temporal model, at a fine spatial resolution from parasitologically confirmed malaria cases and incorporated metrics on healthcare use as well as measures of uncertainty associated with incidence predictions. We then combined the incidence estimates with population maps to estimate clinical burdens and show the benefits of such mapping to identifying areas and seasons that can be targeted for improved surveillance and interventions. Fine spatial resolution maps produced using this approach were then used to target resources to specific local populations, and to specific months of the season. This remote targeting can be especially effective where the population distribution is sparse and further surveillance can be limited to specific local areas.

An increasing number of countries at the margins of the endemic range of malaria are moving towards elimination of the disease^1^,^2^,^3^. As countries transition to controlled low-endemic malaria transmission settings, challenges arise in terms of how the disease burden is quantified and how to produce robust operational malaria cartography to guide control and elimination strategies^4^,^5^,^6^. A primary reason for this is that the disease tends to cluster in hotspots and is also hard to detect parasites at low levels of transmission in the general population using the current diagnostic methods^7^. The traditional snapshot prevalence surveys are not suitable for tracking changes in burden in low malaria transmission, because malaria cases are usually sporadic, susceptible to changes in climate, ecology, population movements or intervention coverages^8^,^9^. As countries in the malaria pre-elimination phase focus on improvement of surveillance and health information systems^10^,^11^, the measurement of incidence (rather than prevalence) based on passive and active surveillance represents a cheaper (compared to investing in active surveys) and potentially important evidence base for mapping transmission, to facilitate the targeting of interventions^12^,^13^.

Using passive surveillance data for mapping malaria requires that health facility cases are parasitologically confirmed via rapid diagnostic tests (RDTs) or microscopy and are complete both spatially and temporally^14^. However, these data are usually incomplete^14^, and thus, approaches are required that adjust for health facility utilisation and under-reporting^15^,^16^,^17^,^18^. Although incidence coupled with information on health care access has been mapped at a coarse resolution in low malaria transmission settings^17^,^18^, there is no robust quantification of incidence at fine spatial resolution from passive surveillance data to guide malaria programmes in controlled low-endemic transmission settings. Where malaria has been eliminated recently, for example, in Turkmenistan, Reunion Island, Mauritius and Tunisia, the elimination programmes relied on the simplified mapping of cases or hand drawn maps (see WHO elimination case studies^19^). These were difficult to produce logistically and update rapidly, were not consistent across large areas and did not benefit from the kind of geographic information system (GIS) integration with population maps^20^ that is available today to estimate burden for planning or effective resource allocation during elimination efforts. Other previous attempts at quantifying malaria risk in low-endemic malaria transmission areas have used ecological niche modelling approaches using presence and absence of malaria cases to predict probability of a case^21^ or using regression tree methods^22^,^23^. Incidence was not estimated directly using these approaches, which also did not incorporate the spatial and temporal dependencies in the data, or measures of uncertainty associated with predictions^24^.

Here, we propose a flexible modelling framework for fine spatial and temporal mapping of *P. falciparum* malaria incidence in elimination settings. A bivariate model specification based on cases aggregated at district level was used to inform predictions of incidence at fine spatial resolution and across multiple time periods. The main goal was the prediction of a time-series of monthly malaria incidence at a fine spatial resolution with complete coverage based on incidence measured at health facilities (i.e., representing a facility catchment, with the equivalent of a coarse spatial resolution), supported by fine resolution remotely sensed spatio-temporal covariates, and incorporating uncertainty. The fine spatial resolution prediction should help pre-elimination programmes to allocate resources effectively to the population most in need, maximising public health impact.

## Overview of analytical framework for mapping of malaria incidence at fine spatial resolution

**Figure 1:**
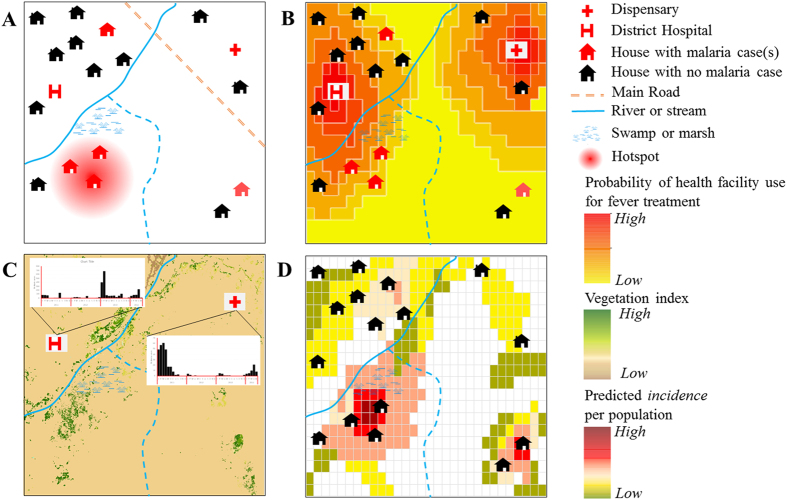
Generalised mapping of fine scale *P. falciparum* incidence in low transmission settings. (**A**) Shows an example landscape. Certain areas such as swamps are breeding sites for anopheles mosquitoes that sustain malaria transmission resulting in focal transmission areas (hotspot with infected houses). Often hotspots are located in areas far from health facilities. (**B**) The probability of seeking treatment at health facilities by population located far from health facility catchment area reduces with distance in addition to other socio-demographic factors. With infected people generally having low immunity in a low-endemic malaria transmission settings, fever onset is likely to be rapid once infected, leading to presentation at health facilities. However some can be missed, such as the house in the lower right corner. (**C**) Assembled cases over time at facilities combined with remotely sensed data from satellites (vegetation) showing potential risk areas. The vegetation index map was obtained from Moderate Resolution Imaging Spectrometer (MODIS) satellite imagery (http://modis.gsfc.nasa.gov/) and map created in ESRI ArcGIS 10.2 software (http://www.esri.com/software/arcgis/). Satellite data cover large geographic areas and are useful in predicting risk in areas with no sampled data. **(D**) Mapping from health facility data coupled with environmental covariates leads to a gridded fine spatial resolution map of predicted incidence for each month, highlighting only populated areas (coloured squares) that can be targeted for interventions and active surveillance.

**Figure 2:**
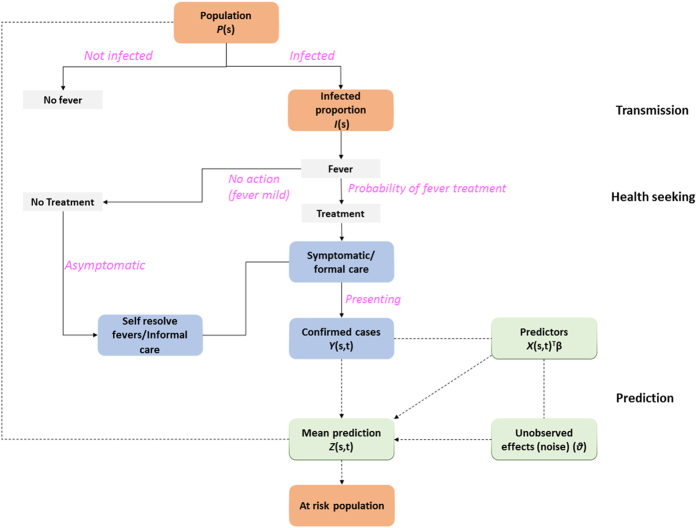
Generalised schematic representation of data flow and analysis for prediction of incidence. Technical details of the modelling framework at prediction stage are provided in the methods section.

A framework for mapping malaria incidence at a fine spatial resolution in low transmission settings is presented here (Figs 1 and 2) and technical details are provided in the methods section. Mapping malaria in low transmission settings is a challenge, since as incidence drops, transmission concentrates in hot spots and “hotpops” (populations that maintain malaria transmission)^25^ (Fig. 1b). It is challenging to identify hotpops operationally because only a subset of febrile individuals may seek treatment at formal (health facilities) and informal (drug shops and drug vendors) centres^26^. As shown in Fig. 2, some mild fever cases, representing a proportion of people infected may self-resolve^26^,^27^. In low malaria transmission settings, immune status plays an important role in treatment seeking patterns^8^. For instance, the onset of fever in low-immunity populations may lead to presentation at peripheral health centres, while populations highly exposed (hotpops) are less likely to seek treatment^28^. The presenting fevers must be confirmed via parasitology for effective case management before treatment based on guidelines from WHO T3 (test-treat-track)^29^. Current evidence suggests an increasing use of diagnostics in health facilities under this initiative^30^.

Data from health facilities provide a means of detecting hotspots of transmission (Figs 1 and 2 for cases presenting at health facilities)^31^,^32^. These data have the advantages of being reported on a continuous basis at a national level providing a temporal signal that captures the seasonal variability of malaria in low transmission settings^33^. For example, cases seen at a health facility can be aggregated by month and used to produce monthly maps of malaria incidence. However, some febrile cases outside health facility catchments may be missed as shown in Fig. 1(c). Thus, using health facility data for mapping malaria incidence requires an adjustment for healthcare use, both in the public and private sectors^34^,^35^. At a national level, such information can be obtained from geo-located nationally representative household surveys such as the Demographic and Health Surveys (DHS) or Malaria Indicator Surveys (MIS)^16^,^36^. Quantifying the probability of health facility use in both public and private health facilities (for example, Fig. 1c) is potentially beneficial in identifying the population not covered by health care systems. Estimating the probability of health facility use combines the behavioural aspect of fever treatment for malaria from household surveys and distance or travel times to a health facility modelled using landscape characteristics such as land cover, urbanisation and elevation^16^,^37^. Although an assumption can be made for treatment seeking behaviour to nearest facility based on distance^38^,^39^, this should be evaluated in different transmission settings^40^. Distance-decay curves are then modelled based on the logistic regression between the binary outcome of fever treatment from household survey and travel times^37^. Probability of use is estimated based on the regression parameters and used as an adjustment to population seen at peripheral health facilities^16^.

The final phase uses Bayesian modelling techniques combined with environmental variables (predictors) that support transmission and malaria case numbers (Fig. 1(c)) to produce a gridded fine spatial resolution map of malaria incidence (Fig. 1(d)). Technical details of Bayesian modelling framework used in this study are provided in the methods section. In general, both static (only spatial) and space-time covariates can be assembled. Advances in satellite remote sensing enable assembly of both climatic (rainfall) and ecological (vegetation) covariates that can be matched with case data from health facilities. These support prediction of incidence at unsampled locations with matching environmental conditions^21^,^41^,^42^. The Bayesian inference method harnesses the spatial and temporal dependencies (covariance) in the observed malaria cases and their relations with environmental predictors while incorporating measures of uncertainty into the output maps of incidence. The generalised linear mixed class of models are used to connect observed data to environmental predictors, making use of spatial and temporal covariances, and incorporating a random error, since the model is never perfect^24^. The combination of the model parameters relating to covariates, model intercept, spatial and temporal covariance, random effects or noise and prior information about the disease builds a model used for computation. The computational modelling is checked via a validation subset data and by comparing the mean predictions (Fig. 2) from modelling to the observed data. In practice, other goodness of fit statistics such as deviance are also estimated^43^. Uncertainty in the model may relate to error sources and affect confidence interval estimates on incidence. These stem from uncertainties observed in case data, environmental variables affecting malaria transmission, health care utilisation and in the Bayesian modelling parameters related to prediction. The confidence interval estimates associated with incidence predictions may be useful in planning and directing resources to populations as well as guiding future surveys to reduce uncertainties in estimates. Incidence estimates are then combined with population maps^20^ to estimate populations at risk at any level (last stage in Fig. 2). At district level, these can be used to categorize high, medium, and low transmission districts for operational and planning purposes. The intersection of a population map with the incidence map may also show “hotpops” that can be targeted for surveillance which may unearth asymptomatic infections.

### Case study: Mapping seasonal *P. falciparum* incidence in Namibia

The modelling framework outlined above was demonstrated in an application to northern Namibia to produce continuous spatial and temporal predictions of *P. falciparum* malaria incidence at 1 × 1 km spatial resolution. Namibia’s current malaria strategy aims to achieve a national case incidence of less than 1.0 per 1000 population by 2017^44^. Assessing the burden of malaria is useful in guiding future surveillance and targeting of malaria interventions. Transmission occurs in the endemic northern regions, considered to contribute almost the entire malaria burden in Namibia^44^,^45^. Therefore, the case study here was restricted to these regions (Fig. 1 in supplementary information).

*P. falciparum* case data, from both public (90.1%) and private (9.9%) health facilities, were obtained from the Namibia National Vector-borne Diseases Control Programme (NVDCP) for 29 months from January 2012 to May 2014 (supplementary information). Healthcare utilisation in Namibia was estimated from the probability of health facility attendance for fever treatment described elsewhere^16^. In brief, travel times were calculated between health facilities and clusters from a national representative survey (the Malaria Indicator Survey (MIS)) and used to derive a distance-decay curve characterizing utilization of health facilities with journey times. Several factors were considered while deriving the travel times including different modes of travel (motorised and non-motorised), land use and land cover characteristics, and type of road. Travel speeds were assigned separately to these factors. The probability of health facility attendance for fever treatment was then modelled using logistic regression based on assumption of using the nearest health facility. The output gridded probability map (similar to Fig. 1b) was combined with a gridded population map and a regional facility reporting rate to derive a population-weighted surface of health facility attendance to provide the denominator in modelling incidence (Fig. 2). The gridded population map was obtained from an online population mapping database (WorldPop^20^) at 100 m spatial resolution and projected forward to 2012, 2013 and 2014 using United Nations national population growth estimates for each year^46^. Modelling incorporated precipitation and vegetation covariates that matched the malaria case data in space and time (dates) (supplementary information for a complete description of covariates). These included monthly precipitation estimates from the Tropical Applications of Meteorology using Satellite data (TAMSAT) and ground based observations^47^ and vegetation index (enhanced vegetation index (EVI)) from the Terra-Moderate Resolution Imaging Spectroradiometer (MODIS) sensor^48^. These covariates were standardized prior to analysis by centering on the mean and dividing by the standard deviation.

The outputs were fine spatial resolution monthly maps of estimated *P. falciparum* incidence in northern Namibia. The data and spatially matched covariates were used in a Bayesian model to produce continuous maps of incidence in each 1 × 1 km grid square in northern Namibia. The Bayesian framework used a linear combination of covariates, malaria cases, and included spatial and temporal effects at the computation stage (see full model parameter specification in supplementary information). In applying the modelling framework, health facility data were aggregated to the district level while uncertainty maps were produced with predictions at a fine spatial resolution. Missing data points were treated as ‘NA’ predicted by the model. Model goodness-of-fit was checked using the deviance information criterion (DIC) and marginal likelihood. A Pearson correlation was used to compare the predictions to observed data using a 20% (*n* = 70 health facilities) validation sample. Other model parameters including the spatial variance, and spatial range were estimated. The predicted incidence estimates were then categorised into three zones of low, medium and high priority districts for operational and planning purposes. The population living in different risk categories was also estimated by extracting total counts after reclassification.

## Results

### The predicted malaria incidence and the clinical burden

**Figure 3:**
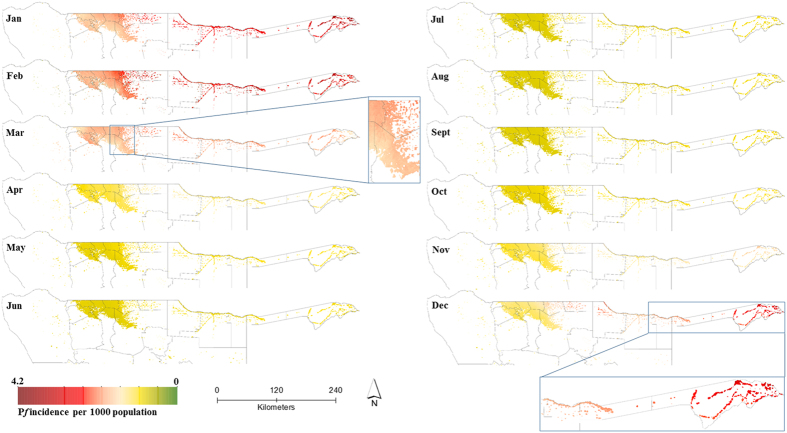
Spatio-temporal maps of predicted mean monthly incidence of *P. falciparum* per 1000 population in northern Namibia in areas with population density greater than 0.01 people per km^2^. The maps were created in ESRI ArcGIS 10.2 software (http://www.esri.com/software/arcgis/). Malaria incidence peaked in the December to April period and was lowest in May to October. The maps suggest less spatial variation between May and October. Accompanying uncertainty maps are included in the supplementary information. Data were from 29 months (from January 2012 to May 2014) based on confirmed malaria cases (*n* = 20,689 cases) from 322 health facilities in northern Namibia.

**Figure 4:**
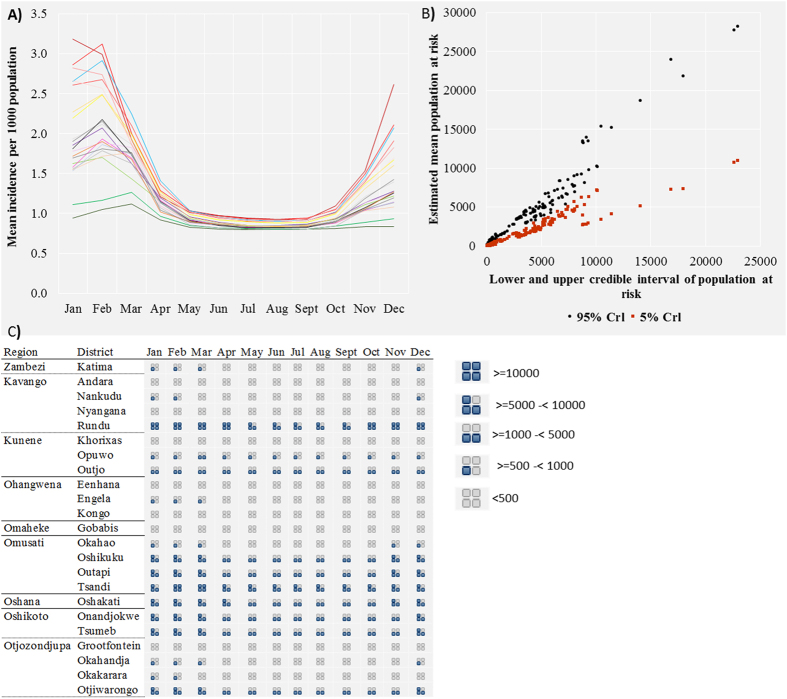
(**A**) The modelled seasonal pattern of incidence of *P. falciparum* per 1000 population by month in 22 northern districts in Namibia. Modelling results suggest that incidence varied by district and month, but was lowest between May and October. (**B**) Scatterplot showing the estimated mean population at risk of *P. falciparum* malaria in Namibia in 2014 and the Bayesian credible intervals based on predicted incidence by district and month. The credible intervals (*Crl: Bayesian credible interval)* were wider where the estimated mean population at risk was more than 10000 but closer to the mean where population at risk was <10000. (**C**) The estimated population at risk of *P. falciparum* malaria in Namibia in 2014 by district and month. Population estimates are based on the worldpop dataset (http://www.worldpop.org.uk/) for Namibia. *P. falciparum* incidence estimates were modelled in a Bayesian framework from assembled malaria cases between 2012 and 2014 and incorporating space-time covariates.

*P. falciparum* malaria incidence in northern Namibia exhibited strong seasonality, and Fig. 3 maps the predicted incidence on a 1 × 1 km grid by month, while Fig. 4(a) shows the variation in predicted incidence at the district level. Figure 4(b) shows a scatterplot with 95% credible interval estimates of population at risk at the district level, while Fig. 4(c) displays the estimated population at risk at the district level by month. The incidence of *P. falciparum* peaked in January and February and was lowest between May and October, with the greatest risk in regions bordering Angola and Zambia in Zambezi, Ohangwena and Kavango. The mean predicted incidence per 1000 population in Zambezi was 3.19 [95% Crl 2.90–3.77] in January and 3.00 [2.91–3.89] in February; and 2.70 [2.50–2.99] in January and, 2.70 [2.51–3.00] in February for Kavango. The mean predicted incidence at a fine spatial resolution for the entire period of data was <1.0 per 1000 population for the majority of the northern regions in Namibia except for Ohangwena region where it was 1.13 [0.26–1.89]. At the 1 × 1 km grid square level, the mean predicted incidence for the entire northern region was 0.86 [0.29–1.45] compared to a crude (non-modelled) incidence estimate of 4.6 cases per 1000 population.

Table 1 shows the estimated case burden by region. Based on the modelled mean incidence for the 29 month period, the majority of the population lived in areas with less than 1.0 case per 1000 population (Table 1). An estimated 76.1% of the population resided in areas with an incidence of 0.5 to 1.0 per 1000 population compared to 17.7% in areas with less than 0.5 cases per 1000 population. Finally, 6.2% lived in regions where mean incidence was estimated to be >1.0 per 1000 population, mainly in Zambezi and Kavango. The two regions combined constitute approximately 18% of the population of northern Namibia (Table 1).

For operational purposes, the Namibia NVDCP has already used the output maps of malaria incidence presented here in stratifying and deciding on appropriate response according to operational strata. In terms of stratification, the highest risk was in districts in Zambezi (Katima, Andara,), Okavango (Nyangana, Rundu, Nankudu) and one district in Ohangwena (Kongo). These districts were categorised as zone 1 districts, requiring concerted effort to reduce malaria incidence to less than 1 case per 1000 population. Zone 2 districts (Groofontein, Okakerara, Otjiwarongo, Tsumed, Onadjokwe, Eenhana, Engela, Oshikuku and Outapi) and zone 3 districts (Gobabis, Opuwo, Khorixas, Outjo, Okahao, Oshakati and Tsandi) should be targeted for elimination starting preferably with any focal transmission areas in these districts. A map showing these priority districts is included in the supplementary information.

Table 2 lists the estimated parameters of the fitted bivariate spatio-temporal model for incidence. In terms of prediction at a fine spatial resolution, the space-time covariates were significant predictors compared to the district level aggregate covariate (precipitation). Although the impact of rainfall on *Anopheles* mosquitoes is well studied^49^,^50^, this direct effect was not assessed here. The correlation parameter, related to the cross-spatial covariance model specification, was close to one. The posterior mean of the variance of the spatial process at pixel level was 0.31 (95% credible interval 0.28–0.36) while the posterior mean of the noise parameter was 1.45 (1.41–1.49) suggesting that the latter (residual component) explained more of the variation in incidence. The model was improved by including mean rainfall estimates at the district level (DIC 43610.76, marginal log-likelihood −38114.92, model complexity 123.24) compared to when excluded (DIC 43820.90, marginal log-likelihood −38170.38, model complexity 117.05). This suggested a justification for using the covariate (mean rainfall estimates) at the district level. Model parameters were also more precisely estimated when spatial covariance that model spatial dependencies in the data were included (DIC 47558.99, marginal log-likelihood −23911.46, model complexity 34.14 by excluding spatial effects). The mean square error (MSE) was 1.02 and RMSE 1.0, based on a 20% validation sample selected randomly. The Pearson correlation was 0.66 with an actual coverage probability of the prediction interval as 94.3% based on nominal coverage at 95%.

## Discussion

As progress is made on shrinking the malaria map^51^,^52^, the focus shifts to providing a strong evidence base to target vectors and parasites and maximise impact at the margins of malaria transmission. Malaria elimination is not only significant to health and economic development in vulnerable populations but also beneficial to other sectors^53^,^54^,^55^. Here, we have outlined a methodological framework to support this goal using passive surveillance data from public and private health facilities to model malaria incidence seasonally and at a fine spatial resolution in low-endemic settings. By aggregating health facility data to operational administrative unit levels, while carrying out predictions at fine spatial resolution, the methodology can be generalizable to other low transmission settings and to other infectious diseases. This improved mapping from passive surveillance data can help inform not only the spatial and temporal targeting of interventions, but also reactive case detection in low transmission settings. The demonstration in Namibia highlights the feasibility of the approach in producing accurate and spatially detailed incidence estimates over the course of a year that can be readily updated as new surveillance data are produced. The use of these maps by the NVDCP in Namibia for operational strategies already is testament to their value.

In general, the predicted incidence in our Namibia case study was low (average predicted incidence per 1000 population for the 29 months period was less than 1.0). *P. falciparum* incidence in this period was anticipated to be lower than that predicted in 2009^18^, owing to increased malaria control and interventions since 2009. In addition, an estimated 6.2% of the population lived in areas where case incidence was greater than 1.0 per 1000 population and this was mainly in regions bordering Angola and Zambia in Kavango and Zambezi. It is possible that some of the malaria cases reported in these regions come from Angola or Zambia, or as a result of Namibians travelling to higher risk areas in these countries. Population movement remains a challenge for the NVDCP in Namibia^23^. An additional observation is that seasonal peaks of incidence occur early in the year coinciding with the rainy period in Namibia that runs from November to April^56^,^57^. Estimates from modelling suggested that risk remains in other regions in northern Namibia, although with a case incidence <1.0 per 1000 in a sparsely distributed population. Aridity limits transmission along the Atlantic and the southern regions of Karas, Hardarp and Erongo in the Namib Desert and the Kalahari Desert (see Fig. 1 supplementary information for a map of Namibia showing these regions) while districts in zone 2 and zone 3 (Fig. 5 of supplementary information) suggest focalised transmission could be targeted for elimination. Therefore, in terms of malaria control, such fine spatial resolution estimates of incidence could be useful in optimizing the delivery of interventions to specific areas in Kavango and Zambezi.

**Figure 5:**
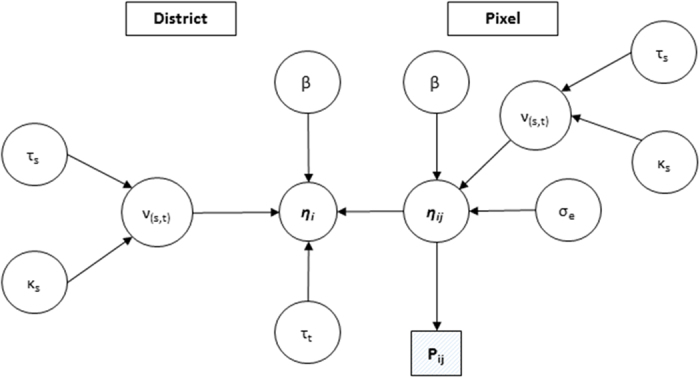
Graphical representation of malaria incidence modelling framework. *P* is the output pixel level incidence at pixel level, η is the specification of linear predictor with spatial effects ν, covariate effects β, residual error σ_e_, and effect of the month (time) τ_t_.

The NVDCP target the use of indoor residual spraying (IRS) using *organochlorines* Dichloro-diphenyl-trichloroethane (DDT) on traditional structures and deltamethrin (*pyrethroid*) on modern houses in endemic regions in northern Namibia^58^. Recent findings suggest current IRS implementation suffers from several problems including planning (number of structures, personnel), poor monitoring and evaluation, logistical (transportation, equipment, insecticide shortages), and operational (geographic reconnaissance)^59^. The current IRS implementation period in Namibia is between September and November. This study suggests priority districts in zone one for targeted spraying, covering the Kavango and Zambezi regions (Fig. 5 supplementary), or blanket coverage in districts being targeted for elimination. Table 1 lists the population at risk applicable to planning of the IRS programme in each region. In addition, results here based on a combination of Figs 3 and 4(a–c) suggest that IRS should ideally be implemented before the start of the transmission season, over a short time period, and covering the relevant geographic areas starting with zone one. Targeted IRS in the relevant geographic areas based on fine resolution maps should theoretically be more economical and sustainable while at same time maximising IRS impact on local vector species^56^,^57^. The same should be applied to targeted coverage of Long Lasting Insecticides Treated bednets (LLIN) in endemic areas. LLIN use in children under the age of five years was estimated at 34% nationally in 2009^45^ when an estimated 0.5 million LLINs were distributed. Gueye *et al*.^58^ reported fluctuating coverage of LLINs in Ohangwena, Kunene and Omusati regions between 2009 and 2011 where LLIN coverage reduced to 21% on average in the three regions. Between 2014 and 2015, just over 0.7 million LLINs were distributed in these endemic regions based on funding from government and the Global Fund to fight AIDS, Tuberculosis and Malaria (GFATM), in order to fill that gap. The predicted incidence map show areas that should be targeted for LLIN coverage rather than universal coverage^60^ in Zambezi (Katima, Andara), Okavango (Nyangana, Rundu, Nankudu) and in Ohangwena (Kongo) to complement the IRS program.

Versions of the Bayesian modelling framework adopted here have been described and applied in other fields^61^,^62^,^63^,^64^ but not in modelling malaria incidence in very low transmission settings using passive surveillance data. Here, we addressed the previous challenges of using HMIS data^14^,^65^,^66^,^67^ by adjusting for health care utilisation in northern Namibia^16^, assumed a static denominator (population only projected forward on annual basis) and carried out predictions at a spatial resolution of 1 × 1 km using remotely sensed covariates linked to case data in space and time, while at same time estimating the uncertainty of prediction. A Bayesian model based approach takes into account the seasonal and environmental effects in adjusting for potential under-reporting as a result of fewer reported cases or missing data^17^. Using ecological and climatic covariates also helps in explained spatial variation at prediction stage in addition to providing information on the climatic suitability of malaria transmission^41^. While both public and private-based health facilities were used in the analysis, only a small fraction of the population in Namibia used the private sector^68^. The rates of health facility reporting were, however, not the 100% required rate even in the regions with most malaria cases in our case study (Zambezi (88%), Ohangwena (90%), and Kavango (88%) as shown in the supplementary information). Reporting rate and classification of cases become important factors in the malaria elimination phase and during prevention of re-establishment^10^,^69^. The model set up relied on a valid spatial-cross covariance function with temporal innovations between aggregated input data and estimates at fine spatial resolution. It is also worth noting that the current improvements in case management in Namibia in which all suspected malaria fevers are diagnosed parasitologically before treatment eliminates the need for adjustment for test positivity rate. Diagnosis is based on Rapid Diagnostics Tests (RDT) and microscopy as recommended by WHO^70^,^71^, although information on number of slides examined is not available. These methods also suffer from uncertainties, for example, in terms of the quality of microscopy and RDTs being inefficient at detecting parasites at low density^72^,^73^. However, use of these case data provided a reliable indicator of the level of malaria incidence at fine spatial resolution, which is potentially useful in pre-elimination settings and not available otherwise.

In extending the methodology to different countries, the methodology outlined can be adapted to other malaria species such as *P. vivax*. Future approaches, however, should incorporate a higher temporally varying denominator^74^ and with spatially varying estimates of health care use within units of analysis. Other possible improvements may be to increase the number of spatially and temporally varying covariates and to assess the effects of static covariates on predictions. Modelling treatment-seeking behaviour can be improved in several ways: firstly, by incorporating uncertainty estimates in the denominator and propagating the quantified uncertainty in estimates of the probability of fever treatment into the incidence modelling and, secondly, by integrating with population movement data to identify the effects of people moving across the border to seek treatment. The effect of population movements could also be combined with incidence maps to delineate self-sustaining transmission *foci* from areas where transmission is supported by population movement.

In conclusion, one of the pillars for assessing the feasibility of malaria elimination is knowledge of the current level of transmission. Here, we demonstrated a method for predicting incidence at a fine spatial resolution for operational purposes in malaria elimination. As more countries transition to low-endemic malaria transmission status (below 5% parasite prevalence), fine spatial resolution mapping is important for identifying the sinks of transmission such as to target interventions. The method is also relevant to countries aiming to scale up interventions, but which do not yet have the active surveillance capacity to adequately capture incidence and transmission burden. Mapping incidence at a fine spatial and temporal resolution improves clinical burden estimates and can be used to target resources to specific local populations. This remote targeting can be especially cost effective where the population distribution is sparse and further surveillance can be limited to specific local areas. The effect of population movements within country and internationally with neighbouring countries remains to be investigated as part of future research.

## Methods

### Bivariate model specification for incidence prediction

The goal was to estimate model parameters and carry out predictions of malaria incidence at fine spatial and temporal resolution. The general framework followed a bivariate modelling framework with a misalignment between prediction and observed data. Here, a joint distribution of incidence at fine spatial resolution using a hierarchical Bayesian bivariate model for *P. falciparum* was used with input data at coarse scales. Note that the methodology is different to univariate conditional autoregressive (CAR) modelling approach used previously for area-level prediction^17^,^18^. The joint framework adopted here relies on a linear combination of independent spatio-temporal processes to construct a valid set of spatial covariances and cross covariances^61^,^63^. Of interest in model parameter space was the correlation parameter related to the cross covariance specification between the data level and at a fine spatial prediction level. A bivariate approach allows for specification of likelihoods at different levels (for example the area-level i.e. the district, the pixel level at fine scale) as a change of support problem^75^,^76^ implemented in R-INLA^77^. Further details on R-INLA are in supplementary information. In brief, Gaussian Markov Random Fields (GMRFs) are used to represent the continuous domain Gaussian Random Field (GRF)^78^,^79^,^80^. The use of GMRFs has a computational advantage over the GRF representation popular with Markov Chain Monte Carlo (MCMC) algorithm due to the sparse nature of the resulting precision matrix of the GMRF representation^80^. For prediction, the interest was not only in estimates (posterior means) at 1 × 1 km resolution, but also the variances or standard deviations associated with the predicted maps.

Starting with a generalized hierarchical regression model specification:


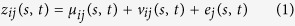


where *z*_*ij*_(*s*, *t*) are realizations of the process for *i*^*th*^ district and *j*^*th*^ pixel, linked to a spatio-temporal structured predictor in an additive way, *μ*_*ij*_(*s*, *t*) arises from a set of local space-time covariates 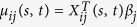 (EVI and precipitation) with *β*_*j*_ coefficients and *e*_*j*_(*s*, *t*) is the estimated measurement error *e*(*s*, *t*) ~ *N*(0, *D*) where *D* is a diagonal matrix 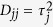. *v*_*ij*_(*s*, *t*) = *Aw*_*ij*_(*s*, *t*), with *w*_*ij*_(*s*, *t*) arising from a zero mean Gaussian Matérn process with covariance cov{*w*(*s*, *t*), *w*(*s*′, *t*′)}≡*C*(*s*, *s*′;*t*, *t*′). The specification of a white noise parameter is useful because variance in incidence is expected to change at fine spatial resolution. The model can be viewed in a hierarchical way with first level specification as *z*(*s*, *t*) ~ *N*(*μ*(*s*, *t*) + *v*(*s*, *t*)), *D*) and at a second-level, the joint distribution of *v*(*s*, *t*) ≡ *v*(*s*_1_, *t*_1_),....., *v*(*s*_*n*_, *t*_*j*_)^'^ equivalent to *MVNorm*(0, Σ_*j*_).

Let the distribution of cases *y*_*i*_ at the district level follow a Poisson distribution with expectation *μ*_*i*_. That is:


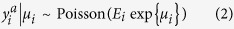


where *E*_*i*_ denotes the proportion of population at risk which is adjusted for a mean healthcare treatment rate and health system reporting rate. Thus, *E*_*i*_ = *Rη*_*i*_ where *R* = *reporting rate x utilisation rate* and log(*λ*_*i*_) = {*β*_0*i*_ + *β*_1*i*_*X*_*i*_(*s*, *t*) + *v*_*i*_(s, t)} with spatial dependence modelled based on centroids of districts via Matérn covariance specification. Note that a CAR model specification could also be applied. The projected denominator population at risk is allowed to vary by year based on the UN population growth rate^46^, but not by month. The vectors of latent variables of the underlying counts 
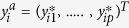
 are then assumed to follow 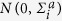 distribution.

The second specification of responses was assumed to be Gaussian with


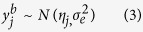


where 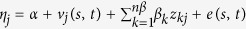 where *α* in the intercept, *v*_*j*_(*s*, *t*) is spatial field specification, on variable *u*, the *β*_*k*_ are the coefficients for the covariates and *e* represents the residual error effects.

Given the above specification from equations 2 and 3, let 
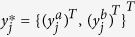
 denote vector of latent variables underlying response at location *j*. It is assumed that 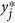 follow a multivariate normal with block covariance matrix 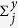
81, generalised for bivariate specification as:


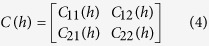


where *h* is a separation distance, Euclidean and


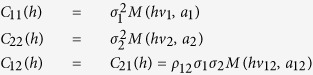


With *C*_11_(*h*) and *C*_22_(*h*) expressing the covariance of the two univariate processes and *C*_12_(*h*) the cross covariance, *ρ*_12_ is the correlation coefficient of the spatial process, with *v* and *a* parameters of the Matérn covariance (*M*) related to the smoothness and scaling respectively. The desired property of the cross covariance (Σ) is that it is positive definite, that is, *a*'Σ*a* ≥ 0. The implementation in R-INLA use a stochastic partial differential equation (SPDE) representation with solution a GF with Matérn covariance is given by^82^ *C*(⋅) = *σ*^2^{2^*v*−1^Γ(*v*)}^−1^(*a*||*h*||)^*v*^*k*_*v*_(*a*||*h*||); where *k*_*v*_ is the modified Bessel function of the second order kind and *h* is the Euclidean distance while *σ*^2^ is the marginal variance. *a* is the scaling parameter, with *a* 0, while *ν* is the Matérn smoothness parameter as defined above and is linked to the variance *σ*^2^ through *α* = *ν* + *d*/2 where *d* is the spatial dimension. *α* = 2 For *d* = 2 and *v* = 1. The marginal variance is then given by *σ*^2^ ≈ 1/4*πk*(*s*)^2^*τ*(*s*)^2^. The scaling parameter relates to the spatial range *r* of the model via *r* ≈ 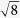/*k*(*s*) with an initial model approximation *r* = 1/5 of the spatial domain. Note that the parameters of SPDE are allowed to vary spatially thus the range parameter approximation is in the local context rather than a global range as it would apply in a stationary case.

The generalised model then takes the form,


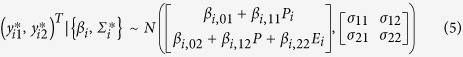


with *β*, *σ*, Σ parameters as explained in preceding sections with precipitation *P* and vegetation *E* as covariate effects.

The joint density of interest is given by;


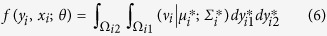


where 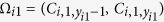, 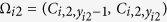, and 
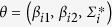
 denoting model parameters (Fig. 5 for full model parameters).

Bayesian specification is therefore completed by assigning prior distributions for *β*_*i*_ ~ *N*(1, 100) (non-informative priors for intercepts and covariates), the parameters of the spatial-temporal fields were defined on the precision matrix of the SPDE defined as *Q* = *T*(*K*^2^*CK*^2^ + *K*^2^*G* + *GK*^2^ + *GC*^−1^*G*)*T* where *T* = *diag*(*τ*(*s*)) and *K* = *diag*(*k*(*s*)) with **s** defined as locations of GMRF representation. The internal representation in R-INLA requires prior specification on *τ* and *k* such that;


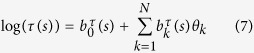



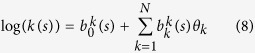


with 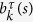 and 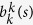 were determined internally from spatial basis functions, while informative Gaussian priors set on *θ* following Ingebrigtsen *et al*.^80^. Thus, *v* ~ *N*(0, *Q*(*k*,*τ*)), log τ ~ *N*(0,1) for equation 7, log *k* ~ *N*(0,1) for equation 8 were used. The model likelihood was computed via the Newton-Raphson method^83^,^84^ where a log-likelihood function ℓ(*θ*; *x*) with *θ* as unknown parameters is maximised with Η(*θ*) as the matrix of the second derivative known as the Hessian matrix. For computational purposes, we checked and ensured that the Hessian was positive-definite (for convergence) by starting with a small *h* value parameter (h = 1e–10 and tolerance = 1e–20). Lastly, weakly informative inverse gamma priors were used for the white noise or residual error parameters *D* ~ *IG*(1, 1*e*–05).

Several statistical parameters were used to assess model fit predictive ability and how well the specified model fitted the data. A validation sample comprising of 20% of case reports (*n* = 70) of the dataset was selected randomly and aggregated at district level. The remainder of the data were used as a training set to estimate model parameters. Firstly, model calibration (statistical consistency) and sharpness (concentration) were assessed using the probability integral transform (PIT) and the conditional predictive ordinate (CPO), a leave-one-out cross-validation approach in which an estimate is validated based on the fitted model and the remaining data only^85^,^86^. The PIT histogram should be a standard uniform distribution for a perfect model where the observed and predicted values are the same^85^. Secondly, estimates of the mean square error (MSE) that quantifies the discrepancy between observed and the predictions, the root mean square error (RMSE) which assess overall performance in the map, Pearson correlation and standardised residuals from the posterior mean were calculated. Pearson’s correlation coefficient was used to compare the predictions (district level after aggregation) to the observed values and scatter plots were produced.

### Ethics Approval

University of Southampton number: 17263.

## Additional Information

**How to cite this article**: Alegana, V. A. *et al*. Advances in mapping malaria for elimination: fine resolution modelling of *Plasmodium falciparum* incidence. *Sci. Rep.* **6**, 29628; doi: 10.1038/srep29628 (2016).

## Supplementary Material

Supplementary Information

## Figures and Tables

**Table 1:** Population count estimate by malaria incidence classification by region in the northern regions of Namibia.

Region	Estimated population 2014	Estimated population count (%) at different malaria incidence		
		<0.5	0.5 to 1.0	>1.0
Zambezi	89,110	15,033 (16.9)	69,611 (78.1)	4,466 (5)
Kavango	188,279	42,216 (22.4)	86,051 (45.7)	60,012 (31.9)
Kunene	70,971	0 (0)	70,863 (99.8)	108 (0.2)
Ohangwena	272,544	111,928 (41.1)	143,536 (52.7)	17,079 (6.3)
Omaheke	104,389	37 (0.0)	104,352 (100)	0 (0)
Omusati	276,893	94,568 (34.2)	182,305 (65.8)	20 (0.0001)
Oshana	166,673	7,203 (4.3)	159,470 (95.7)	0 (0)
Oshikoto	184,969	1,154 (0.6)	171,485 (92.7)	12,330 (6.7)
Otjozondjupa	180,952	0 (0.0)	179,163 (99)	1,789 (1)
Total	1,534,781	271,719 (17.7)	1,168,524 (76.1)	94,537 (6.2)

A map showing regions (place names in the table) is included in the supplementary information as Fig. 1).

**Table 2:** Parameters of the fitted bivariate Bayesian spatial-temporal model of incidence using aggregated cases at district level and supported by space–time covariates.

Parameter	Mean	Standard Deviation	5%	Median	95%	
Intercept	I_D_	−1.0369	0.1600	−1.3004	−1.0369	−0.7733
	I_p_	−1.5049	0.0620	−1.9362	−1.5047	−1.0738
Precipitation	β_P1_	−0.0025	0.0202	−0.0409	−0.0025	0.0360
EVI	β_P2_	−0.0817	0.0275	−0.1271	−0.0817	0.0311
Precision for month	τ_t_	0.8953	0.0908	0.2988	0.5456	3.0182
Precipitation	β_D1_	0.1339	0.0145	0.1101	0.1339	0.1578
Spatial range	r_P_	0.2470	0.0319	0.2005	0.2435	0.3048
	r_D_	2.1118	0.4666	1.3440	2.1122	2.8869
Correlation	Corr(P, D)	0.9353	0.0312	0.8848	0.9344	0.9882
Gaussian white noise	σ_ep_^2^	1.4549	0.0244	1.4150	1.4545	1.4957
Spatial Variance	v_p_	0.3145	0.0258	0.2750	0.3124	0.3610
	v_D_	1.6665	0.0081	1.6530	1.6666	1.6799

District level aggregated estimates of precipitation were used, but not for EVI where only 1 × 1 km mean estimates were used. Two intercepts were included in the bivariate model and spatial variance and spatial range parameters were also estimated.
