# Erratum: Advances in mapping malaria for elimination: fine resolution modelling of *Plasmodium falciparum* incidence

**DOI:** 10.1038/srep32908

**Published:** 2016-09-14

**Authors:** Victor A. Alegana, Peter M. Atkinson, Christopher Lourenço, Nick W. Ruktanonchai, Claudio Bosco, Elisabeth zu Erbach-Schoenberg, Bradley Didier, Deepa Pindolia, Arnaud Le Menach, Stark Katokele, Petrina Uusiku, Andrew J. Tatem

Scientific Reports 6: Article number: 2962810.1038/srep29628; published online: 07 13 2016; updated: 09 14 2016

In this Article, the columns in Table 2 are misaligned. The correct Table 2 appears below:

## Figures and Tables

**Table 2:** 

Parameter		Mean	Standard Deviation	5%	Median	95%
Intercept	I_D_	−1.0369	0.1600	−1.3004	−1.0369	−0.7733
	I_p_	−1.5049	0.0620	−1.9362	−1.5047	−1.0738
Precipitation	β_P1_	−0.0025	0.0202	−0.0409	−0.0025	0.0360
EVI	β_P2_	−0.0817	0.0275	−0.1271	−0.0817	0.0311
Precision for month	τ_t_	0.8953	0.0908	0.2988	0.5456	3.0182
Precipitation	β_D1_	0.1339	0.0145	0.1101	0.1339	0.1578
Spatial range	r_P_	0.2470	0.0319	0.2005	0.2435	0.3048
	r_D_	2.1118	0.4666	1.3440	2.1122	2.8869
Correlation	Corr(P, D)	0.9353	0.0312	0.8848	0.9344	0.9882
Gaussian white noise	σ_ep_^2^	1.4549	0.0244	1.4150	1.4545	1.4957
Spatial Variance	v_p_	0.3145	0.0258	0.2750	0.3124	0.3610
	v_D_	1.6665	0.0081	1.6530	1.6666	1.6799

