# Supplementary material for: Advances in mapping malaria for elimination: fine resolution modelling of Plasmodium falciparum incidence
Source: Sci Rep. 2016 Jul 13;6:29628. doi: 10.1038/srep29628 (PMC4942778; doi:10.1038/srep29628)
Supplement: Supplementary Information [file srep29628-s1.doc]

**Supplementary information**

**Advances in mapping malaria for elimination: fine resolution modelling of *Plasmodium falciparum* incidence**

Victor A Alegana^1*^, Peter M Atkinson^1,2,6^, Christopher Lourenço^1,4^, Nick W Ruktanonchai^1^, Claudio Bosco^1^, Elisabeth zu Erbach-Schoenberg^1^, Bradley Didier^4^, Deepa Pindolia^4^, Arnaud Le Menach^4^, Petrina Uusiku^5^, Stark Katokele^5^, and Andrew J Tatem^1,3^

**S1.0 Malaria pre-elimination in Namibia**

Namibia declared its ambition to eliminate malaria by 2020 after reducing the burden significantly in the last 10 years through sustained control ^1,2^. Namibia’s current malaria strategy aims to achieve a national case incidence of less than 1.0 per 1000 population by 2016 ^3^. Namibia is ranked as an upper-middle income country following substantial economic growth with an annual GDP of approximately 4.8% from 2000 to 2013 ^4^. However, there still exist large inequalities in income (estimated Gini coefficient of 0.6 in 2010^5^) and the majority of the rural population is poor. The country is sparsely populated (approximately 3 persons per square km) with an estimated 65% living in the northern nine regions that are predominantly rural ^6^. The risk of malaria is constrained by the climate which varies from arid to semi-arid. The Namib Desert stretches along the Atlantic while the Kalahari Desert runs along the border with Botswana in the southeast. The larger and more sparsely populated south is considered either ‘*malaria-free*’ (< 1.0 cases per 10,000 population) or supporting highly focal, very low transmission intensity. *Anopheles arabiensis* remains the dominant malaria vector in Namibia with 97% of malaria cases due to infection with *Plasmodium falciparum* ^3^.


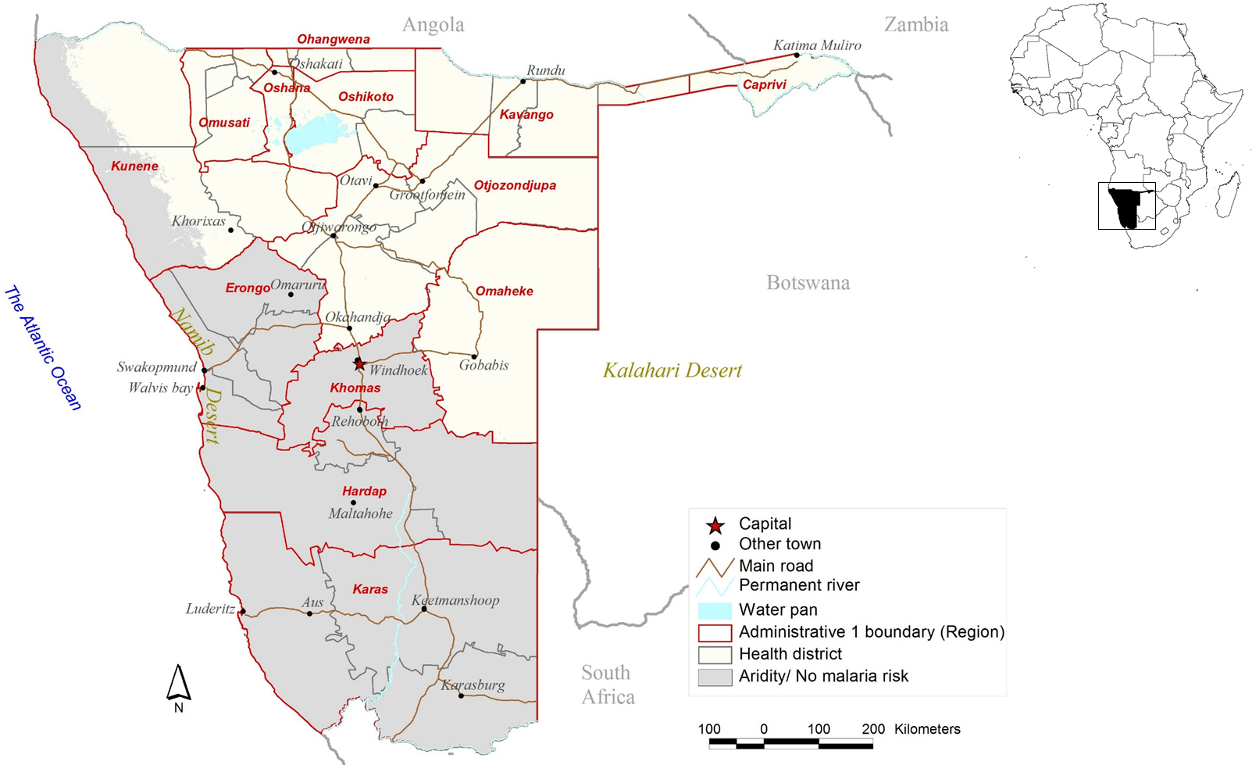


Figure 1: Map of Namibia showing administrative 1 boundaries and limits of *P. falciparum* risk (light yellow) at health administrative areas (districts) and the locations of the major urban areas. The grey regions have no malaria risk mainly due to extreme aridity. The map was created in ESRI ArcGIS 10.2 software (http://www.esri.com/software/arcgis/)

**S2.0 Summary of data**

In total, there were 322 health facilities in northern Namibia comprising of 290 public and 32 private based facilities. The public based facilities were managed by Ministry of Health and Social Services (MHSS), mission and non-governmental organisations and a few managed by the Police or the Ministry of Defence. A complete description of the health facility list in Namibia can be found here ^7^.

*S2.1 Malaria case data*

From the assembled data, the number of confirmed *P. falciparum* cases observed at health facilities in northern Namibia was 3,299 in 2012, 4,745 cases in 2012, and 12,945 cases in 2014 (Table 1). This represented a national-level crude incidence of 2.4 cases per 1000 population in 2012, 3.1 cases per 1000 population in 2013 and 8.4 cases per 1000 population in 2014, before adjusting for health facility utilisation. The overall average heath facility reporting rate was 24 out of the expected 29 monthly case reports (Table 1) with the highest rates were in Oshikoto (26.8) and the lowest in Oshana (19.7).

**Figure 2:** Plot of the number of cases assembled by month, matched with spatio-temporal covariates (precipitation (mm) and enhanced vegetation index (EVI)). Precipitation was obtained from gridded monthly Tropical Applications of Meteorology using SATellite (TAMSAT) data combined with rain gauge observations. EVI was downloaded from MODIS.

**Table 1**: Summary of data for the northern regions in Namibia

| **Region** | **Number of health facilities** | | **Estimated mean probability of HF utilisation** |  | **Number of confirmed P*f* malaria cases** | | | **Average reporting rate^1^** |  | **Estimated population** | | |
| --- | --- | --- | --- | --- | --- | --- | --- | --- | --- | --- | --- | --- |
|  | **Public** | **Private** |  |  | **2012** | **2013** | **2014** |  |  | **2012** | **2013** | **2014** |
| Zambezi | 31 | 3 | 0.60 |  | 1,274 | 2,564 | 1,965 | 25.29 |  | 85,138 | 87,102 | 89,110 |
| Kavango | 62 | 3 | 0.49 |  | 1,019 | 1,454 | 10,210 | 25.38 |  | 179,886 | 184,035 | 188,279 |
| Kunene | 28 | 3 | 0.27 |  | 131 | 50 | 9 | 22.97 |  | 67,808 | 69,371 | 70,971 |
| Ohangwena | 33 | 2 | 0.63 |  | 196 | 236 | 202 | 26.23 |  | 260,395 | 266,401 | 272,544 |
| Omaheke | 17 | 2 | 0.34 |  | 13 | 4 | 46 | 20.00 |  | 99,735 | 102,035 | 104,389 |
| Omusati | 51 | 4 | 0.55 |  | 418 | 161 | 200 | 25.87 |  | 264,550 | 270,651 | 276,893 |
| Oshana | 19 | 6 | 0.57 |  | 49 | 48 | 43 | 19.72 |  | 159,243 | 162,916 | 166,673 |
| Oshikoto | 24 | 3 | 0.45 |  | 81 | 43 | 97 | 26.78 |  | 176,724 | 180,800 | 184,969 |
| Otjozondjupa | 25 | 6 | 0.36 |  | 46 | 85 | 88 | 20.65 |  | 172,886 | 176,873 | 180,952 |
| **Total** | **290** | **32** |  |  | **3,299** | **4,745** | **12,945** | **21.22** |  | **1,466,366** | **1,500,184** | **1,534,781** |

1. Average reporting rate across the region represents mean number of months out of expected 29 months with reported data. Missing values were recorded as NAs.

*S2.2 Space-time covariates*

Only covariates that matched the case data in space and time were considered for analysis. These were precipitation and vegetation. The enhanced vegetation index (EVI), a measure of photosynthetic activity ranging from 0 (no vegetation) to 1 (complete vegetation), was downloaded from Moderate-resolution Imaging Spectroradiometer (MODIS) imagery at a spatial resolution of 1 by 1 km (<http://modis.gsfc.nasa.gov/>) ^8^. The enhanced vegetation index was obtained from MODIS/Terra monthly product defined as:

$$EVI=2.5\times\frac{NIR-RED}{NIR+\left(6\times RED-7.5\times BLUE\right)+1}$$

where NIR (near-infrared), red and blue refer to the reflectance in the corresponding parts of the electromagnetic spectrum (with corrections for atmospheric effects – Rayleigh and ozone absorption). EVI quantifies the difference between the visible red spectrum and NIR. In the red spectrum, there is strong absorption of incident radiation by chlorophyll compared to the NIR where scattering is exhibited ^9^. Thus, the index is also dependent on soil-surface to vegetation reflectance in addition to atmospheric effects. These effects are adjusted using the visible blue reflectance (to correct for aerosol effects in the visible spectrum) and a canopy adjustment factor ^10,11^. The index is, therefore, most sensitive in areas with large biomass. However, an adjustment for aerosol effects, which is absent in the more common normalized difference vegetation index (NDVI), potentially makes it a better index.

We assembled gridded mean monthly average precipitation estimates from the Tropical Applications of Meteorology using SATellite (TAMSAT) ^12^. TAMSAT combines thermal infrared data from the geostationary Meteosat (meteorology) satellite acquired approximately every 15 minutes with ground based observations from over 4000 stations to predict rainfall amount. The basic algorithm first estimates a 10 day rainfall amount (dekad) and aggregates three dekads to obtain a monthly product at approximately 4 km spatial resolution. The dekadal optimisation algorithm assumes a linear relationship between the duration of convective clouds (cold cloud duration in hours) and the amount of rainfall observed ^13^. Validation is conducted by comparing pixel level predictions to ground observations.

Previous work in Namibia suggested that these two covariates EVI and Precipitation are useful predictors of malaria ^14,15^. In Sub-Saharan Africa, the publication by Noor et al. ^15^ list covariates for each country after an extended statistical selection procedure. This can be used as a guide in future for countries in pre-elimination in SSA. A statistical procedure could also be used to select a minimum set of space-time covariates matching case data (aiming for parsimony) that have a biologically plausible relationship with malaria transmission ^14^. This is also useful in avoiding statistical over-fitting ^16^. Although temperature plays a key role in transmission a Temperature Suitability Index (TSI) based on the survival of vectors and monthly temperature effects on the duration of sporogony was not selected. The TSI is constructed using long-term monthly temperature time series and represented on a scale of increasing transmission suitability, from 0 (unsuitable) to 1 (most suitable) with most areas in northern Namibia suitable. Note that accessibility (travel time to health facilities), urbanisation and Land cover were used in deriving the probability of health facility utilisation.

**S3.0 Methods**

*S3.1 INLA SPDE/GMRF*

A hierarchical spatio-temporal model, implemented via stochastic partial differential equation (SPDE) method ^17,18^, was used for joint estimation at pixel level. The method in summary uses a *Gaussian Markov Random Function* (GMRF) by approximating a set of spatial-temporal random functions with a weighted sum of spatio-temporal basis representations, for instance, on $x$ as:

$$x(s,t)=\sum_{k=1}^{n} \phi_{k}(s,t)x_{k}$$

Where each basis function is the Kronecker product $\phi_{k}(s,t)=\phi_{i}^{s}(s)\phi_{j}^{t}(t)$in space and time. The advantage of using the GMRF representation stems from its Markovian properties that result in sparse precision matrices that are easier to deal with computationally. However, the link to the continuous domain Gaussian Function (GF) is a solutions to a space-time SPDE of the form ^18,19^

$$\left(k(s)^{2}-\Delta\right)^{\alpha/2}\left(\tau(s)x(s,t)\right)=w(s,t)$$

Where $\left(k(s)^{2}-\Delta\right)^{\alpha/2}$a differential operator, $k$ is the scaling parameter, $\Delta$ is the Laplacian, $\alpha$controls the smoothness of the realization, $\tau$controls the variance, $x(s,t)$is the weight vector and $w(s,t)$is the Gaussian white noise. A non-stationary model was implemented by allowing the scaling parameter of the SPDE to vary spatially on locations of centroids of districts. The SPDE generates a precision matrix of the form where is the precision in the spatial domain and in temporal domain corresponds to a one dimensional AR(1) model. The AR(1) model for a Gaussian vector $x_{i}$is defined as $x_{i}=\rho x_{i-1}+\varepsilon_{i}$where the coefficient and $\varepsilon\sim N(0,\tau^{-1})$, $i=2,...,n$ ^20^. where C, G_1_ and G_2_ are sparse matrices.

*S3.2 R-INLA code*

A summarised R-INLA code for spatial-temporal joint modelling of malaria incidence given confirmed cases, expected population using health care services and some environmental covariates is presented below. Note that only the model specification is presented. Other routine sections of the code such as reading in data files, standardising covariates are not included for brevity.

*S3.3 Internal and external model validation checks*

Figure 4 shows validation plots extracted from modelling while figure five shows the standard deviation by month at fine spatial resolution. Model calibration (statistical consistency) and sharpness (concentration) were assessed using the probability integral transform (PIT) and the conditional predictive ordinate (CPO), a leave-one-out cross-validation approach in which an estimate is validated based on the fitted model and the remaining data only ^21,22^. The PIT histogram should be a standard uniform distribution for a perfect model where the observed and predicted values are the same ^21^.

**
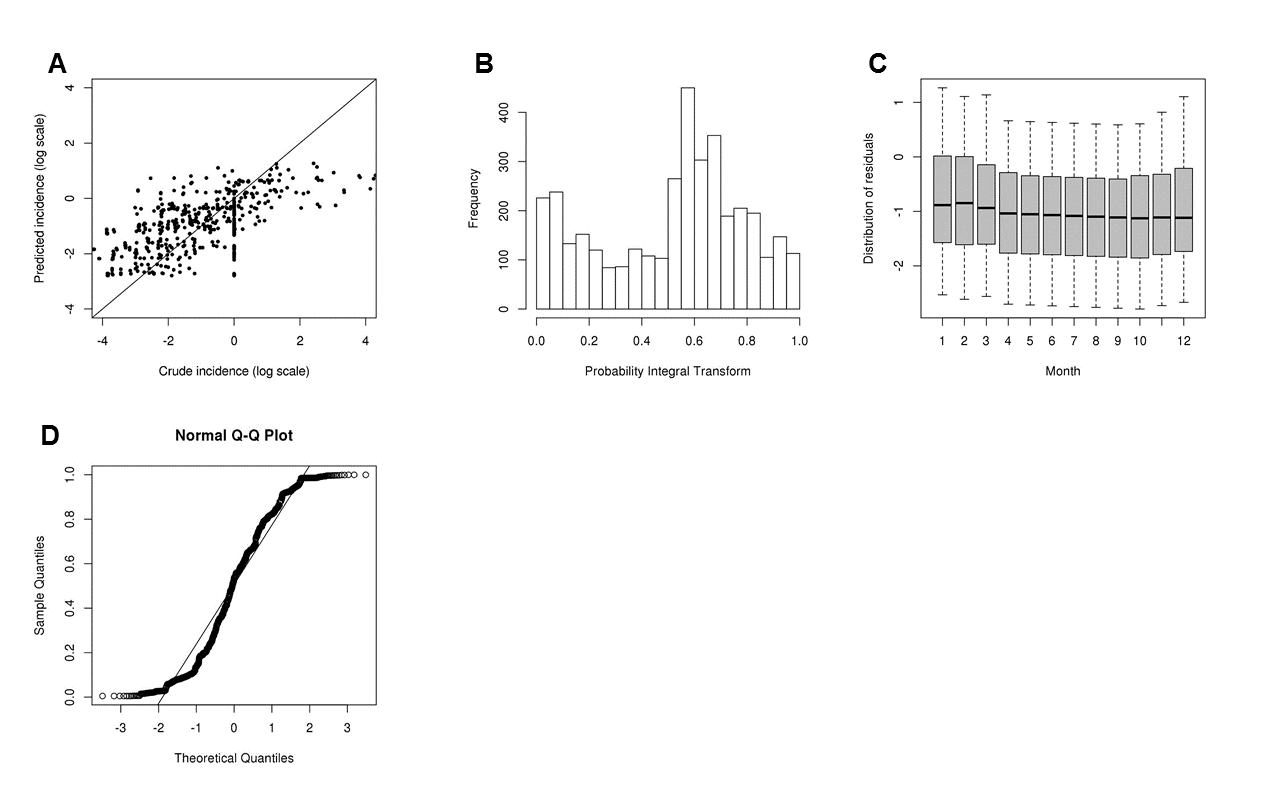
**

**Figure 4**: **Model validation panel**. **A)** A scatterplot between the estimated crude malaria incidence per 1000 population at district level and the mean prediction based on a 20% subset. **B)** Calibration check based on the probability integral transform (PIT) histogram with bins. The histograms for this application indicated lack of perfect model calibration. U-shaped histograms indicate underdispersion while the inverse U-shape indicates an overdispersed predictive distribution. **C)** The distribution of residuals from the posterior mean by month showing some seasonality in the first three months of the year. **D)** The quantile plot of the posterior density of the standardised residuals.


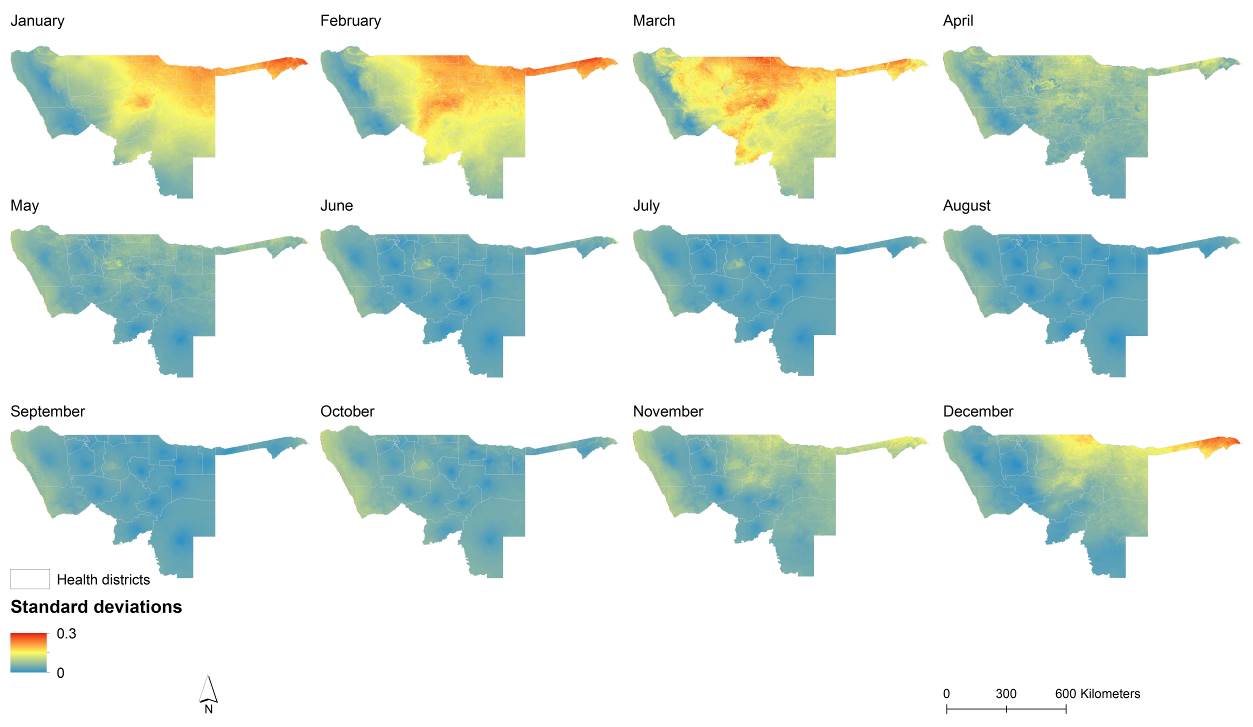


**Figure 5**: Spatial maps showing variation of standard deviation around the predicted mean incidence (2012-2014) of *P. falciparum* per 1000 population in northern Namibia estimated using the bivariate Bayesian spatio-temporal model. The maps were created in ESRI ArcGIS 10.2 software (http://www.esri.com/software/arcgis/)


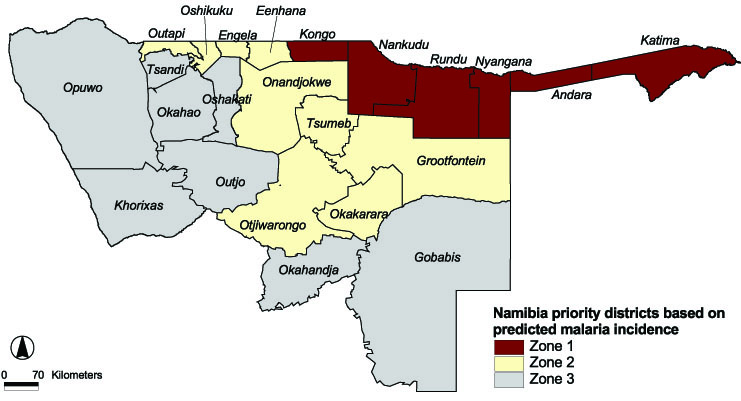


**Figure 6**: District level operational map showing priority districts for malaria intervention. Zone 1 with mean incidence >1.4 per 1000 population, zone 2 with mean incidence >1.2 -< 1.4 and zone 3 with incidence <1.2 per 1000 population. We suggest zone 2 and zone 3 be targeted for elimination with increased interventions to reduce burden in zone 1 to less than one case per 1000 population. The map was created in ESRI ArcGIS 10.2 software (http://www.esri.com/software/arcgis/)
